# Highly efficient evaluation of diffusion networks in Li ionic conductors using a 3D-corrugation descriptor

**DOI:** 10.1038/s41598-019-51585-z

**Published:** 2019-10-22

**Authors:** Arthur France-Lanord, Ryoji Asahi, Benoît Leblanc, Joohwi Lee, Erich Wimmer

**Affiliations:** 1 Materials Design SARL, 92120 Montrouge, France; 2 Toyota Central R&D Laboratories, Inc, Nagakute, Aichi 480-1192 Japan; 3 Present Address: Department of Materials Science and Engineering, Massachusetts Institute of Technology, Cambridge, Massachusetts 02139 USA

**Keywords:** Computational methods, Batteries

## Abstract

A highly efficient computational approach for the screening of Li ion conducting materials is presented and its performance is demonstrated for olivine-type oxides and thiophosphates. The approach is based on a topological analysis of the electrostatic (Coulomb) potential obtained from a single density functional theory calculation augmented by a Born-Mayer-type repulsive term between Li ions and the anions of the material. This 3D-corrugation descriptor enables the automatic determination of diffusion pathways in one, two, and three dimensions and reproduces migration barriers obtained from density functional theory calculations using nudged elastic band method within approximately 0.1 eV. Importantly, it correlates with Li ion conductivity. This approach thus offers an efficient tool for evaluating, ranking, and optimizing materials with high Li-ion conductivity.

## Introduction

In search for high-performance Li ion conducting materials, the height of migration barriers for ionic diffusion is a fundamental criterion for the screening and ranking of new candidates. The commonly used nudged elastic band (NEB)^1^ method using quantum mechanical calculations can provide accurate values for barrier heights, but this approach is not well suited for automated screening, because it requires prior knowledge of the diffusion paths, and it is computationally demanding. Similarly, molecular dynamics simulations using quantum mechanics or classical forcefields are very useful for detailed investigations but are not appropriate for high-throughput calculations on large number of candidates. In practice, high-quality forcefields are often not readily available for all systems of interest. Thus, alternative approaches that are computationally efficient, robust, sufficiently accurate, and suitable for automation are desirable.

To this end, Nakayama *et al*.^2^ proposed an efficient way to estimate the Li pathway by using a model potential based on empirical bond valence (BV) relations between the energy and the interionic distance of Li and oxygen. The three-dimensional migration pathway can be determined by a percolation algorithm. Application of this approach to 14 olivine-type oxides shows a good correlation in the predicted barrier heights compared with density functional theory (DFT) results obtained with NEB calculations, but deviations are relatively large, and the absolute values are systematically overestimated. Recently, Zimmermann *et al*.^3^ introduced a scheme combining DFT-calculated electrostatic potential data with finite-size ion models. The minimum energy path is then determined using an elastic-band algorithm. This method results in excellent linear correlation with NEB-calculated barrier energies, but suffers some drawbacks: (i) if there are no available reference NEB barrier energies, the finite-size ion model cannot be optimized. In this case, the authors recommend using a full-sphere model with the ion’s Wigner-Seitz radius, which results in less accurate, although still acceptable, predicted barriers; (ii) the model does not predict absolute values of the barrier energies; and (iii) as standard NEB, this approach still requires the user to input initial and final states. Hence, there is a need for improvements.

In this paper we present a method that predicts absolute barrier heights for Li ion diffusion in oxides and sulfides while remaining fully automatic and computationally efficient. The present method consists in (i) the construction of an effective potential experienced by diffusing Li ions, called 3D-corrugation descriptor, and (ii) its topological analysis to determine the descriptor barrier energies interpreted as the Li-ion diffusion barriers. This approach maps out all diffusion pathways of a given structure without prior information of the topology of the system and it computes the related diffusion barriers using information from a single DFT calculation without the need for multiple NEB calculations. Thus the proposed 3D-corrugation descriptor enables an efficient screening and ranking of Li ion conducting materials in terms of the descriptor barrier energies.

## Results

In order to assess the 3D-corrugation descriptor’s ability to capture the correct physics of Li diffusion in complex compounds as well as variations in barrier energies as a function of systematic chemical modifications, we have evaluated its performance on a set of olivine-type compounds and on thiophosphates. The olivines used here have the general composition Li *MX* O_4_, where *M* is an atom from groups 2, 3, 13, or from the lanthanide series, and *X* is either a pnictogen or an atom from the carbon group. These crystals all belong to the *Pnma* space group (orthorhombic lattice).

Jalem *et al*.^4^ reported NEB barrier energies on a set of 32 of these compounds. Using the 3D-corrugation descriptor, we have computed descriptor barrier energies and diffusion pathways in all but one of these structures, a Ce(III) oxide, which is notoriously difficult to model with standard DFT due to the strongly correlated Ce *f* electron^5^. In addition, no guidelines were provided in the original reference^4^ to include any self-interaction or on-site correction; we could therefore not reproduce this result.

**Figure 1:**
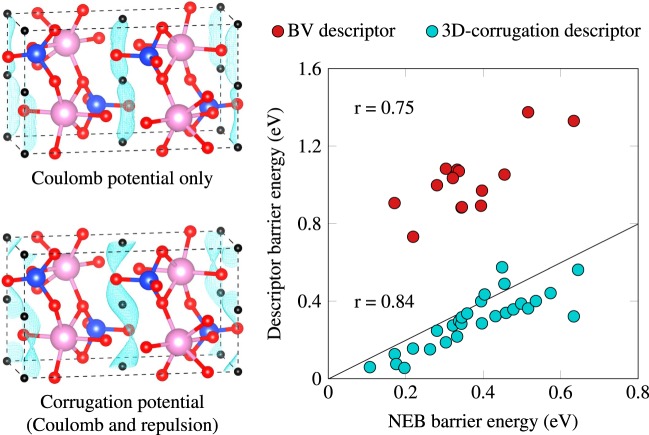
Performance of the 3D-corrugation descriptor on a set of olivines. Left: 1D corrugation pathway (in Cyan) in LiInSiO_4_ as obtained from the electrostatic (Coulomb) potential alone and the one including the repulsive potential. The black spheres indicate equilibrium Li sites. Right: performance of the 3D-corrugation descriptor in predicting NEB barrier energies for a set of olivines, compared to the BV descriptor. Also reported are the Pearson correlation coefficient values *r*.

In the left panel of Fig. 1, we illustrate the 1D corrugation pathway in LiInSiO_4_ identified by using either the Coulomb potential alone, or including the repulsive part. As one can see, the short-range Pauli repulsion from neighboring negatively charged species (here O) is clearly underestimated in the Coulomb case, resulting in the Li sites incorrectly located at the saddle points of the 1D corrugation. On the other hand, including the repulsive potential gives the corrugation energy minimum properly at the Li equilibrium sites, and leads to the correct topology of the diffusion pathways. In addition, the descriptor barrier energy including repulsion (0.287 eV) agrees well with the NEB barrier energy (0.396 eV).

The performance of the BV and the 3D-corrugation descriptors for NEB barrier energies smaller than 0.7 eV, most relevant with high ionic conductivity, is presented in the right panel of Fig. 1. The BV descriptor systematically overestimates the NEB barrier energy, Nakayama *et al*. assigned this systematic shift to the neglect of local relaxation during Li jumps^2^. The 3D-corrugation descriptor produces better absolute values as well as a better correlation. In addition, the Pearson correlation coefficient is high (*r* = 0.84), which leads to a *p*-value smaller than 10^−5^, indicating very strong evidence of an excellent correlation with the NEB results. The *p*-value associated with the BV descriptor results is larger (~0.001), but still provides evidence of a good correlation with NEB data. Finally, the root-mean-square error (RMSE) of the descriptor barrier energies for 3D corrugation with respect to the NEB reference data is of 0.108 eV, almost exactly the same as for the fcc and bcc Li_2_O and Li_2_S training set.

**Figure 2:**
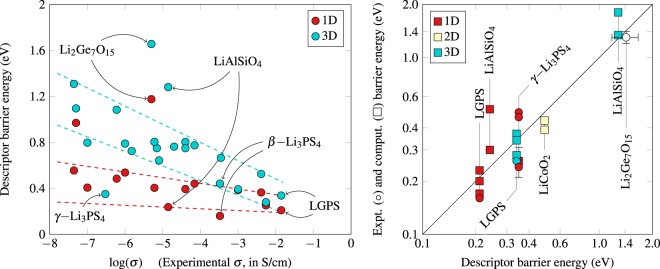
Left: Correlation between experimental Li ion conductivity at room temperature^6,7^, and computed 1D- and 3D-corrugation descriptor barriers. Dashed lines are guide for the eye. Right: correlation between the descriptor barrier energy and the experimental^10–16^ (°) and theoretical^17–24^ (W) activation energy, for several outliers labelled in the left panel, as well as for LGPS. The color corresponds to the dimensionality of the diffusion pathway: red for 1D, beige for 2D, cyan for 3D, and white when the dimensionality is not known.

Since the height of the diffusion barrier for Li ions is a fundamental criterion for ion conductivity, one would expect that there exists a correlation between the diffusion barriers obtained from the present 3D-corrugation descriptor and the experimental ionic conductivity. To probe this correlation, we have taken a set of 22 compounds from the list compiled by Sendek *et al*.^6^ and Phuc *et al*.^7^ where experimental conductivity data have been published, and computed the 3D-corrugation descriptor. The correlation between these two quantities is shown in Fig. 2, and all values are listed in Table S1 of the Supplementary Information.

Despite the fact that the ionic conductivity depends on a number of factors other than the height of diffusion barriers, such as the concentration of Li ions active at a given temperature, the presence of vacancies, cooperative diffusion of groups of Li ions, and coupling with phonon modes of the host lattice^8^, the present descriptor barrier energies show a surprisingly good linear relation with the logarithm of conductivity. It is gratifying to see that the present descriptor correctly identifies Li_10_GeP_2_S_12_ (LGPS) and Li_10_SnP_2_S_12_^9^ as the compounds with the highest Li ion conductivity. Overall, the 3D corrugation seems to be a more robust descriptor than the 1D corrugation. This is reasonable, since the experimental species are polycrystalline and the effective rate-limiting diffusion path may form a 3D corrugation network connecting at the grain boundaries. In other words, diffusion pathways in all three dimensions are involved in the transport of Li ions. In fact, *p*-values extracted from Pearson correlation coefficients for both 3D- and 1D-corrugation descriptor barriers show that the experimental conductivities correlate weakly with the 1D results (*p* = 0.041 > 0.01), and much more strongly (*p* = 0.004) with the 3D energies.

As one can see in the left panel of Fig. 2, there are several outliers in the low ionic conductivity range (*σ* < 10^−5^ S/cm), including Li_2_Ge_7_O_15_, LiAlSiO_4_, and *γ*− Li_3_PS_4_. For these compounds – as well as for LGPS, which deserves special attention due to the anisotropy of its diffusion pathways and for its exceptionally high conductivity – we have investigated in greater details the correlation between corrugation descriptor barriers and reported experimental^10–16^ and theoretical^17–24^ activation energies. The results are presented in the right panel of Fig. 2: we report a very satisfying agreement between these two quantities. In particular, the 1D (0.21 eV along the *c* axis) and 3D (0.34 eV, in the (*ab*) plane) corrugation descriptor barriers calculated for LGPS are in excellent agreement with a range of reported values obtained through various approaches, including standard DFT-NEB^18^ (respectively 0.23 eV and 0.37 eV), site-averaged DFT-NEB^17^ (0.20–0.21 eV, and 0.34 eV), *ab initio* molecular dynamics^19^ (0.17 eV and 0.28 eV), and solid-state nuclear magnetic resonance^11^ (0.16 eV and 0.26 eV). The diffusion channels obtained using the 3D-corrugation descriptor are illustrated in Fig. S1 of the Supplementary Information. Finally, we have also included a layered compound, LiCoO_2_, in order to probe 2D diffusion. Our descriptor successfully predicts a 2D hopping mechanism, with an associated descriptor barrier energy of 0.48 eV, in good agreement with DFT-NEB calculations (0.39 eV^23^, 0.44 eV^24^).

## Discussion

We have reported a method enabling the efficient screening of ion conducting materials. The method requires as input a periodic structure with given atomic positions and delivers as output a corrugation potential experienced by migrating Li ions in one, two, and three dimensions, *i.e*. a 3D-corrugation descriptor. In contrast to NEB calculations, the present approach does not require any *a priori* knowledge of diffusion pathways, it can be automated, and it is computationally fast. A typical NEB calculation requires to optimize the supercell geometry of many images, which leads to a large number of DFT calculations (typically < 100) on several images (usally less than ten). In contrast, the present method requires only a single DFT calculation of the unit cell, as well as the evaluation of the classical repulsive potential and corrugation analysis, which are computationally highly efficient. While estimating the speedup compared to NEB is therefore challenging since it will depend on the system size and the complexity of the diffusion pathway, one can safely assume at least two orders of magnitude speedup in computational time, and perhaps more importantly, the suppression of any human time devoted to generating NEB starting and ending configurations based on assumptions on the diffusion pathway. As a comparison, Zimmermann *et al*.’s method^3^ should lead to almost exactly the same speedup as our method, since the only significantly expensive part of the algorithm is a DFT calculation of the Coulomb potential. The authors provide a conservative estimate of the speedup versus NEB of four orders of magnitude, which also applies to the present approach. Furthermore, NEB calculations are sometimes difficult to converge while the present descriptor approach is computationally robust. As shown in the present work, the 3D-corrugation descriptor provides excellent estimates of diffusion barriers and correlates with Li ion conductivity. Hence, this approach is well suited for high-throughput screening and optimization of large number of compounds.

While the accuracy in predicting barrier heights is satisfactory, namely 0.1 eV, there is an opportunity to improve the correlation with Li ion conductivity (*cf*. left panel of Fig. 2. The present method does not take into account the dependency of the corrugation descriptor on the Li ion concentration nor the dependency on temperature. For example, some Li ions may be strongly bound and do not contribute to the Li ion conductivity. This leads to two possible strategies to improve the current methodology. One possibility is to identify the strongly bound Li ions and optimize the number of Li ions to determine the corrugation potential, *i.e*., in this case, not all Li ions are removed in the DFT calculations but only a subset. Another possibility is to include the temperature effect by performing molecular dynamics simulations, selecting a subset of configurations from the dynamics trajectory, and averaging the corrugation descriptors over those configurations.

On the other hand, to improve the high-throughput performance, the calculation of the electrostatic potential does not have to be accomplished by a self-consistent electronic structure calculation, but could also be derived, for example, from a point-charge model. However, modern electronic structure calculations have become fast and robust enough to avoid the ambiguities and limitations of point-charge models, especially if only single point energy calculations are needed as in the present approach.

While this method has been demonstrated here for the case of Li diffusion in oxides and sulfides, the approach can be extended to other ion conductors, e.g. with protons or other cations such as Na^+^ including crystalline and amorphous systems such as organic polymers. Thus, the 3D-corrugation descriptor presented in this work offers exciting opportunities to explore a large design space for applications such as batteries and fuel cells.

## Methods

It is assumed that the energy of a positive Li ion at a given position in an ionic lattice is dominated by the electrostatic (Coulomb) potential of the surrounding anions, and by the Pauli repulsion at short Li–anion distances. The electrostatic potential as experienced by a *positive* test charge is generated on a three-dimensional grid from a single DFT calculation of the system with all Li atoms removed. This potential is the negative of the Coulomb potential used in electronic structure calculations (*i.e*. the solution of Poisson’s equation) without the exchange-correlation potential. No charge compensation is added to the system, which is equivalent to removing neutral Li atoms. Removing Li^+^ ions and including a compensating background charge has been tested, resulting in an overall shift in the corrugation potential without significantly affecting relative energy differences.

**Figure 3:**
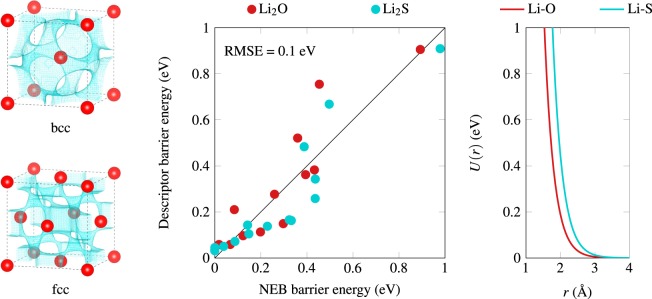
Optimization of the classical repulsive potential. Left: typical migration pathways obtained for the bcc and fcc Li_2_O lattices. Middle: Performance of the algorithm in predicting barrier heights as compared to the NEB reference from Wang *et al*.^26^, for both O and S anionic sublattices. Right: potential energy curves for the optimized Li–O and Li–S repulsive potential.

On each of the grid points, a repulsive potential arising from the neighboring anions, *i.e*. O and S, is added. This classical potential takes the Born–Mayer form^25^:

1 $$U(r)=A{e}^{-\frac{r}{\rho }},$$

where *r* is the distance between a Li grid point and a lattice site (either S or O), and *A* and *ρ* are fitting parameters. We have optimized *A* and *ρ* for Li–S and Li–O interactions in order to match the energy barrier of Li diffusion in S and O fcc and bcc lattices, as a function of the lattice parameter. Comprehensive NEB data have been reported by Wang *et al*.^26^, which we have used as a fitting reference. These barriers correspond to the hopping from a tetrahedral to another tetrahedral site in a bcc lattice, and from a tetrahedral to an octahedral site in an fcc lattice. The optimization process is similar to the one reported in previous work^27^ with a first round using a genetic algorithm in order to efficiently sample the full parameter space, and a second and final one with a standard non-linear least square solver. The performance of the final set of parameters is shown in Fig. 3. As one can see, the optimized set of parameters allows for a satisfying description of the NEB barrier heights, with a RMSE of 0.1 eV. As expected, the repulsive potential for O and S shown in Fig. 3 is consistent with their ionic radii. The optimized parameters, *A* and *ρ*, are given in Table S2 in the Supplementary Information.

**Figure 4:**
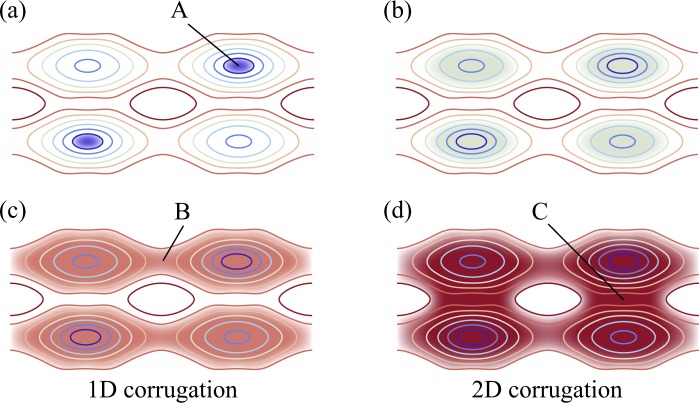
Schematic of determining diffusion pathways. The contour lines represent the corrugation potential. Starting in (**a**) with the lowest value, A, the energy is gradually increased (**b**) until the first percolation at point B is reached. (**c**) The energy difference between the minimum A and the first saddle point B defines a descriptor barrier height for “1D corrugation,” which is associated with the height of the diffusion barrier in that dimension. The process is continued (**d**) until a percolation in a second dimension is obtained at point C defining a descriptor barrier height for 2D corrugation. The third dimension (not shown here) is treated analogously to define a descriptor barrier height for 3D corrugation.

In the topological analysis of the corrugation potential, the 3D grid from the DFT calculations is used and possible diffusion pathways and associated barrier heights are evaluated as follows (*cf*. Fig. 4):

• The lowest value of the corrugation potential is used as a start and a trial energy is raised stepwise;

• For each new trial energy, all grid points are identified whose energy is lower than the trial energy and those grid points are connected;

• Connected points, that are neighbors in the grid, are grouped into one or more subsets;

• Each of the connected subsets is scanned to check whether they traverse the cell in one, two, or three dimensions;

• By raising the trial energy level progressively, one can determine at which energy level 1D, 2D, and 3D percolation occurs;

• The energy difference between the minimum and the energy of first percolation determines the descriptor barrier energy.

This computational procedure defines a 3D-corrugation descriptor and the corresponding descriptor barrier energy fully automatically and efficiently without assuming any diffusion paths in advance.

## Supplementary information

Supplementary Information: Highly efficient evaluation of diffusion networks in Li ionic conductors using a 3D-corrugation descriptor
