# Supplementary material for: Highly efficient evaluation of diffusion networks in Li ionic conductors using a 3D-corrugation descriptor
Source: Sci Rep. 2019 Oct 22;9:15123. doi: 10.1038/s41598-019-51585-z (PMC6805878; doi:10.1038/s41598-019-51585-z)
Supplement: Supplementary file 1 — Supplementary Information: Highly efficient evaluation of diffusion networks in Li ionic conductors using a 3D-corrugation descriptor [file 41598_2019_51585_MOESM1_ESM.pdf]

Supplementary Information: Highly efficient evaluation of diffusion networks in Li ionic conductors using a 3D-corrugation descriptor

Arthur France-Lanord,[†,‡] Ryoji Asahi,[*,¶] Benoît Leblanc,[†] Joohwi Lee,[¶] and Erich Wimmer[†]

†*Materials Design SARL, Montrouge, France*

‡*Current address: Department of Materials Science and Engineering, Massachusetts Institute of Technology, Cambridge, Massachusetts 02139, USA*

¶*Toyota Central R&D Laboratories, Inc., Nagakute, Aichi, 480-1192, Japan*

E-mail: rasahi@mosk.tytlabs.co.jp

1. Computational methods

All *ab initio* calculations were carried out using DFT[1,2] as implemented in the Vienna *Ab initio* Simulation Package (VASP v5.4)[3,4] and integrated in the MedeA®[5] computational environment. Exchange–correlation effects were treated using the generalized gradient approximation (GGA), with the PBE functional developed by Perdew *et al.*[6] The all–electron frozen–core projector augmented wave method (PAW) is employed to solve the Kohn–Sham equations,[7] using plane–wave basis sets with a kinetic energy cutoff of 500 eV. The Brillouin zone integration was carried out with a k-spacing of 0.3 $Å^{-1}$ including the Γ point using the

Methfessel-Paxton scheme with a smearing width of 0.2 eV. The total energy is converged within 10^{-5} eV. These computational parameters ensure that the numerical precision is well within the deviation between energy barriers predicted by the current method and by the NEB calculation.

The repulsion potential was evaluated using the Large Atomic Molecular Massive Parallel Simulator (LAMMPS)[8] code, as implemented in MedeA®. Here the repulsive interaction was truncated using a cutoff distance of 5.0 Å. This choice was made to capture the short-range interactions between Li-ions and the nearest neighboring anions. Note that the LAMMPS calculations do not include any electrostatic terms which are provided by the DFT calculations.

All atomistic models were prepared using the MedeA®[5] simulation environment. All images of atomistic models were produced using VESTA.[9]

2. List of compounds, ionic conductivity, and 1D and 3D corrugation energies reported in Figure 2

Table S1: Experimental room temperature ionic conductivity, σ, from Sendek *et al.*[10] and energy barriers computed with the present corrugation descriptor. The compounds are sorted according to decreasing conductivity.

ID	Compound	σ (S/cm)	log(σ)	1D	2D	3D
3	$Li_{10}GeP_2S_{12}$ (LGPS)	1.40×10^{-2}	-1.854	0.209	0.337	0.337
2	$Li_{10}SnP_2S_{12}$	5.50×10^{-3}	-2.260	0.252	0.279	0.279
33	$Li_7P_3S_{11}$	4.10×10^{-3}	-2.387	0.364	0.437	0.525
1	$LiLaTiO_2$	1.00×10^{-3}	-3.000	0.385	0.385	0.392
32	$Li_7La_3Zr_2O_{12}$	3.50×10^{-4}	-3.456	0.665	0.665	0.665
N/A[a]	$\beta-Li_3PS_4$	3.30×10^{-4}	-3.481	0.160	0.160	0.441
11	$Li_{14}Ge_2V_2O_{16}$	7.00×10^{-4}	-4.155	0.441	0.445	0.774
13	$Li_{148}Ge_{34}W_6O_{40}$	4.00×10^{-5}	-4.398	0.394	0.406	0.803
28	$Li_6BaLa_2Ta_2O_{12}$	4.00×10^{-5}	-4.398	0.749	0.749	0.749
24	$Li_5La_3Bi_2O_{12}$	2.00×10^{-5}	-4.699	0.762	0.762	0.762
35	$LiAlSiO_4$	1.40×10^{-5}	-4.854	0.237	1.277	1.280
25	$Li_5La_3Nb_2O_{12}$	8.00×10^{-6}	-5.097	0.643	0.643	0.643
31	$Li_6SrLa_2Ta_2O_{12}$	7.00×10^{-6}	-5.155	0.749	0.749	0.749
12	$Li_{15}Ge_3VO_{16}$	6.03×10^{-6}	-5.220	0.404	0.433	0.804
7	$Li_2Ge_7O_{15}$	5.00×10^{-6}	-5.301	1.175	1.655	1.655
26	$Li_5La_3Ta_2O_{12}$	1.50×10^{-6}	-5.824	0.724	0.724	0.724
5	$Li_{14}ZnGe_4O_{16}$	1.00×10^{-6}	-6.000	0.537	0.537	0.789
10	$Li_{136}Si_{28}S_{12}O_{160}$	6.00×10^{-7}	-6.222	0.484	1.050	1.083
17	$\gamma-Li_3PS_4$	3.00×10^{-7}	-6.523	0.350	0.350	0.350
14	$Li_3Fe_2P_3O_{12}$	1.00×10^{-7}	-7.000	0.406	0.637	0.797
39	$LiZr_2P_3O_{12}$	5.00×10^{-8}	-7.301	0.969	0.969	1.095
19	Li_3VO_4	4.40×10^{-8}	-7.357	0.555	0.555	1.308

[a]: Experimental data from Phuc *et al.*[11]

3. Diffusion pathways in LGPS computed using the 3D-corrugation descriptor

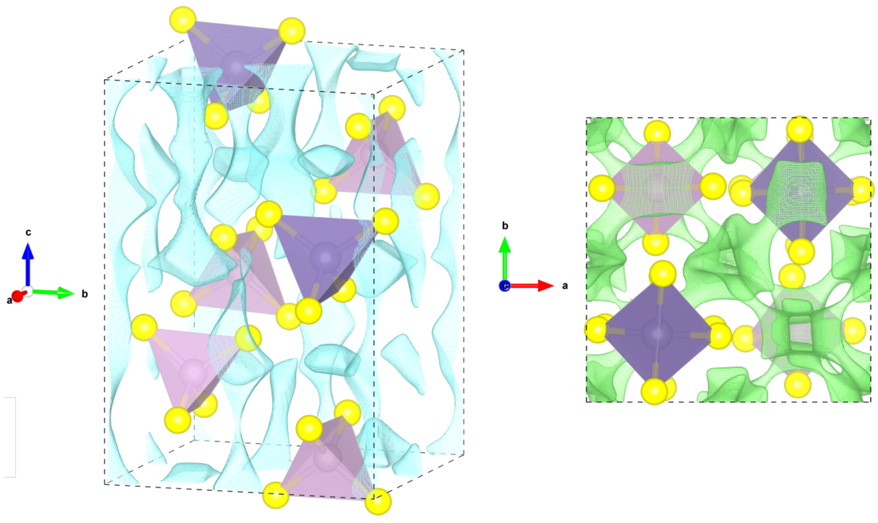

Figure S1: Diffusion pathways in LGPS calculated using the 3D-corrugation descriptor. The 1D corrugation pathway is collinear with the c direction, and the 2D corrugation is coplanar with the (ab) plane.

4. Li–O and Li–S optimized parameters of the Born–Mayer repulsion potential

Table S2: Li–O and Li–S optimized parameters of the Born–Mayer repulsion potential.

ij pair	A_{ij} (eV)	ρ_{ij} (Å)
Li–O	246.97	0.278354
Li–S	512.67	0.283987

References

(1) Hohenberg, P.; Kohn, W. Inhomogeneous electron gas. *Physical review* **1964**, *136*, B864.

(2) Kohn, W.; Sham, L. J. Self-consistent equations including exchange and correlation effects. *Physical review* **1965**, *140*, A1133.

(3) Kresse, G.; Hafner, J. Ab initio molecular dynamics for liquid metals. *Physical Review B* **1993**, *47*, 558.

(4) Kresse, G.; Furthmüller, J. Efficiency of ab-initio total energy calculations for metals and semiconductors using a plane-wave basis set. *Computational materials science* **1996**, *6*, 15–50.

(5) MedeA 2.22, Materials Design, Inc., San Diego, CA USA. 2019.

(6) Perdew, J. P.; Burke, K.; Ernzerhof, M. Generalized gradient approximation made simple. *Physical review letters* **1996**, *77*, 3865.

(7) Blöchl, P. E. Projector augmented-wave method. *Physical review B* **1994**, *50*, 17953.

(8) Plimpton, S. Fast parallel algorithms for short-range molecular dynamics. *Journal of computational physics* **1995**, *117*, 1–19.

(9) Momma, K.; Izumi, F. VESTA 3 for three-dimensional visualization of crystal, volumetric and morphology data. *Journal of applied crystallography* **2011**, *44*, 1272–1276.

(10) Sendek, A. D.; Yang, Q.; Cubuk, E. D.; Duerloo, K.-A. N.; Cui, Y.; Reed, E. J. Holistic computational structure screening of more than 12000 candidates for solid lithium-ion conductor materials. *Energy & Environmental Science* **2017**, *10*, 306–320.

(11) Phuc, N. H. H.; Totani, M.; Morikawa, K.; Muto, H.; Matsuda, A. Preparation of Li_3PS_4 solid electrolyte using ethyl acetate as synthetic medium. *Solid State Ionics* **2016**, *288*, 240–243.
